# De novo emergence, existence, and demise of a protein-coding gene in murids

**DOI:** 10.1186/s12915-022-01470-5

**Published:** 2022-12-08

**Authors:** Jan Petrzilek, Josef Pasulka, Radek Malik, Filip Horvat, Shubhangini Kataruka, Helena Fulka, Petr Svoboda

**Affiliations:** 1 Institute of Molecular Genetics of the Czech Academy of Sciences, Videnska 1083, 142 20 Prague 4, Czech Republic; 2 Present address: Vienna BioCenter PhD Program, Doctoral School of the University of Vienna and Medical University of Vienna, Vienna, Austria; 3 Bioinformatics Group, Division of Biology, Faculty of Science, University of Zagreb, Horvatovac 102a, 10000 Zagreb, Croatia; 4 Present address: Department of Genetics, Yale School of Medicine, New Haven, CT 06510 USA; 5 Current address: Institute of Experimental Medicine of the Czech Academy of Sciences, Videnska 1083, 142 20 Prague 4, Czech Republic

**Keywords:** De novo, Gene, Evolution, LTR, Retrotransposon, CAG, Polyserine, D6Ertd527e, Oocyte

## Abstract

**Background:**

Genes, principal units of genetic information, vary in complexity and evolutionary history. Less-complex genes (e.g., long non-coding RNA (lncRNA) expressing genes) readily emerge de novo from non-genic sequences and have high evolutionary turnover. Genesis of a gene may be facilitated by adoption of functional genic sequences from retrotransposon insertions. However, protein-coding sequences in extant genomes rarely lack any connection to an ancestral protein-coding sequence.

**Results:**

We describe remarkable evolution of the murine gene *D6Ertd527e* and its orthologs in the rodent *Muroidea* superfamily. The *D6Ertd527e* emerged in a common ancestor of mice and hamsters most likely as a lncRNA-expressing gene. A major contributing factor was a long terminal repeat (LTR) retrotransposon insertion carrying an oocyte-specific promoter and a 5′ terminal exon of the gene. The gene survived as an oocyte-specific lncRNA in several extant rodents while in some others the gene or its expression were lost. In the ancestral lineage of *Mus musculus*, the gene acquired protein-coding capacity where the bulk of the coding sequence formed through CAG (AGC) trinucleotide repeat expansion and duplications. These events generated a cytoplasmic serine-rich maternal protein. Knock-out of *D6Ertd527e* in mice has a small but detectable effect on fertility and the maternal transcriptome.

**Conclusions:**

While this evolving gene is not showing a clear function in laboratory mice, its documented evolutionary history in *Muroidea* during the last ~ 40 million years provides a textbook example of how a several common mutation events can support de novo gene formation, evolution of protein-coding capacity, as well as gene’s demise.

**Supplementary Information:**

The online version contains supplementary material available at 10.1186/s12915-022-01470-5.

## Background

*Some are born to sweet delight*

*Some are born to endless night*

*[William Blake, 1863]*

After a gene comes into being, it evolves and lasts for a variable period of time. The term gene historically denotes the basic physical and functional unit of heredity [1] but the definition has been evolving along with advancement of knowledge of genomes and transcriptomes (reviewed in [2]). In traditional gene definitions, a molecule encoded by a gene (an RNA or a protein) has a function. However, proving an absence of a function of a molecule encoded by a putative gene is an impossible task. Furthermore, the evolutionary theory implies that genetic traits emerge purposelessly before their function is established by means of natural selection. Thus, any DNA sequence encoding a defined RNA molecule may be considered a gene.

A distinct category of genes are protein-coding genes, where an RNA carries information for protein synthesis. Protein-coding genes usually evolve through some mechanism involving an existing protein-coding sequence as evidenced by clusters of orthologous groups and conserved protein domains [3, 4]. In fact, many extant protein-coding genes are descendants of genes of the last universal common ancestor [5]. In contrast, long-non-coding RNA (lncRNA) genes in complex eukaryotic genomes often emerge from more-or-less random genomic sequences and have rapid evolutionary turnover [6–8]. Notably, lncRNAs and protein-coding genes are not separately evolving discreet gene classes; protein-coding genes can lose the protein-coding capacity and become lncRNAs [9, 10]. At the same time, the coding potential of cytoplasmic lncRNAs is being probed by scanning ribosomes [11]. LncRNAs may occasionally become bona fide protein-coding genes, particularly when a processed pseudogene integrates into an existing lncRNA unit [12]. However, complete de novo emergence of protein-coding capacity in a lncRNA is rare [13].

Emergence of a novel gene from a random sequence does not require much as cryptic promoters, splice sites, and poly(A) sites emerge in a random sequence by a considerable chance. In addition, functional gene parts can be stochastically distributed across a genome by transposable elements [14, 15]. While amplification of transposable elements threatens genome integrity, they can also move around functional gene parts, such as promoters, enhancers, exons, terminators or splice junctions (reviewed in [16, 17]). A common source of functional genic elements are long terminal repeats (LTRs), identical sequences flanking internal sequences of transposable elements (TEs) and retroviruses. 5′ LTRs serve as promoters while 3′ LTRs provide a functional polyadenylation signal. Significance of functional sequences in LTRs is underscored by the fact that most LTR retrotransposon insertions in mammalian genomes become solo LTRs, which form when homologous recombination recombines out the internal sequence between LTRs [18, 19]. A solo LTR carrying a functional promoter and a poly(A) site is a versatile platform offering several ways how it could shape transcriptional landscape at the insertion site. Indeed, LTRs were often co-opted (exapted [20]) as promoters and exons on hundreds of occasions in the mammalian germline [12, 21] and dozens of cases were documented also in mammalian somatic cells (reviewed in [22]).

A specific feature of the mouse genome evolution has been repeated expansion of the non-autonomous mammalian apparent LTR retrotransposon (MaLR) group, which generated ~ 340,000 insertions [23, 24]. Rodent MaLR elements evolved from ancestral MLT family elements into two families denoted ORR and MT. In the lineage leading to mice, ORR and MT families underwent several amplifications during the last 60 million years, giving rise to specific subfamilies; for example, the MTD subfamily expanded in the mouse genome ~ 40–50 million years ago (MYA) and pre-dated the MTC subfamily, which expended 30–40 MYA [23]. MT elements have oocyte-specific expression and their LTRs often carry a conserved splice donor [12]. Thus, an MT LTR essentially carries the first exon, which can be “plugged in” into an existing gene, or can create a novel transcriptional unit in the genome [12, 21]. In rodent germ cells and early embryos, there are hundreds of protein coding genes and lncRNAs utilizing MaLR-derived promoters and first exons [12]. MTD and MTC LTR insertions, which still function as promoters and first exons could be under positive selection. For example, an MTC subfamily LTR insert in *Dicer1* gene drives oocyte-specific isoform of the protein and is essential for normal oocyte function [25].

We previously reported that an MTD subfamily LTR insertion provided the promoter and the first coding exon in a de novo formed protein-coding gene annotated in the mouse genome as *D6Ertd527e* [12]. Here, we provide an extended analysis of evolutionary history of the *D6Ertd527e* locus in *Muroidea* rodents (mice, rats, gerbils, hamsters, voles, and relatives), and we show that the locus offers textbook example of events occurring during a protein-coding gene “life cycle.”

## Results

### Features of murine D6Ertd527e

**Fig. 1:**
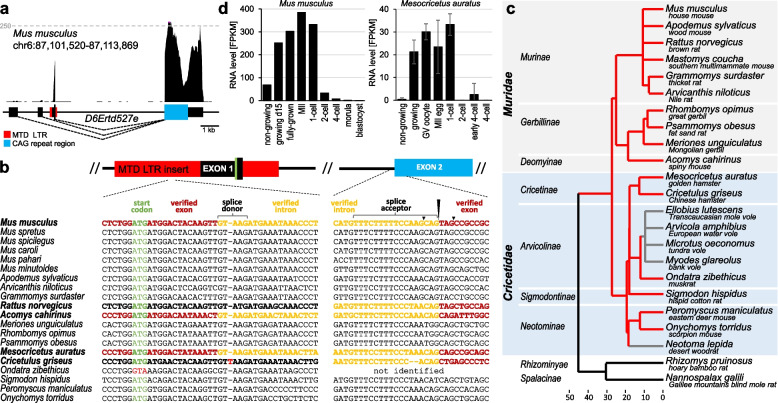
Murine *D6Ertd527e* gene characterization. **a** Scheme of the gene and alternative transcripts in mouse oocytes. Shown is a modified UCSC genome browser snapshot depicting expression in the oocyte, three alternative 5′ exons, and the common 3′ exon. The red rectangle depicts position of the MTD LTR insert providing the dominating oocyte-specific promoter and the first exons. The blue rectangle depicts CDS overlapping with a CAG repeat expansion. Dashed line indicates level of 250 counts per million (CPM) in RNA-seq data from C57Bl/6 fully-grown GV oocytes [27]. **b** Conservation of selected *D6Ertd527e* sequences in species having the MTD LTR insertion. The left alignment of sequences shows an MTD region carrying the AUG codon, the putative coding part of the 5′ exon, and the splice donor. The right alignment of sequences shows the splice acceptor sequence and beginning of the 3′ exon. In bold font are sequences from species where RNA-seq data are available. Colored in brown and yellow are exon and intron sequences, which were validated by RNA-sequencing. In red are depicted two notable mutations. Homologous sequence of the splice acceptor in *Ondatra* was not reliably determined. **c** Adapted timetree [28] of selected rodent species showing basic taxonomic grouping and their phylogenetic relationship. Red lines depict a part of the phylogenetic tree associated with *D6Ertd527e* MTD LTR in extant genomes. Grey lines lead to species, where the MTD LTR insertion was lost by deletion. The timetree was generated by the TimeTree of Life 5 resource [29]. The timescale below the tree is in millions of years ago as approximated by the TimeTree 5 tool. For more precise phylogenetic analysis of muroid species and discussion of divergence dates see [30]. **d** *D6Ertd527e* expression in mouse and hamster oocytes and zygotes. Data were compiled from published RNA-seq data from mouse [31, 32] and hamster [33] samples. Replicates were available for hamster data, *n* = 3, error bars = SD

*D6Ertd527e* was first annotated as an anonymous expressed DNA segment in mice [26] and later identified as a protein-coding gene expressed in mouse oocytes [12]. *D6Ertd527e* is localized in a syntenic intergenic region between *Gfpt1* and *Atrx* genes (Additional file 1: Fig. S1a): in the central part of the chromosome 6 and its gene structure has two remarkable features (Fig. 1a).

First, the main *D6Ertd527e* promoter and the first exon are an exaptation of an MTD LTR insertion [12]. Sequence conservation in *Muroidea* species suggests that the original MTD LTR insert in the common ancestor of mice and hamsters carried a full 5′ terminal exon with an AUG codon (Fig. 1b, c). There are at least two weak upstream promoters with 5′ terminal exons (Fig. 1a), but they do not seem to give rise to protein-coding transcripts in mice [12].

Second, *D6Ertd527e* encodes a serine-rich protein and its coding sequence (CDS) largely evolved from an expanding and mutating (CAG)_n_ repeat. While the (CAG)_n_ repeat expansion and the coding sequence considerably vary among *Muroidea* genomes, the unique 3′ UTR sequence of *D6Ertd527e* is conserved across placental mammals (Additional file 1: Fig. S1b).

Consistent with oocyte-specific expression of MT elements, *D6Ertd527e* is maternally expressed [12]. Expression of *D6Ertd527e* is detectable in non-growing mouse and golden hamster oocytes and the transcript accumulates during oocyte’s growth (Fig. 1d). In both species, *D6Ertd527e* mRNA remains stable until the 1-cell stage but is degraded afterwards (Fig. 1d). Consistent with robust maternal expression, RNA-seq data from mouse organs [34] show the highest transcript level of *D6Ertd527e* in the ovary (Additional file 1: Fig. S1c). RNA-seq suggested a low expression of *D6Ertd527e* in several other organs (testes, intestine, colon, spleen, lung), but none of those transcripts originated from the MTD promoter (Additional file 1: Fig. S1d). Detailed inspection of RNA-sequencing data from the somatic tissues exhibiting low *D6Ertd527e* expression revealed the last exon of *D6Ertd527e* can be rarely spliced with upstream *Gfpt1* exons, forming a rare alternative 3′ end of *Gfpt1.*

### Loss of the MTD promoter of D6Ertd527e during evolution

Analysis of MTD insertions suggested that the MTD LTR promoter of *D6Ertd527e* was lost at least three times during genome evolution in *Cricetidae* (Additional file 1: Fig. S2). Two distinct deletions were observed in genomes of *Neotominae* subfamily. One deletion was found in *Neotoma lepida* (desert woodrat) and the other one in a deermouse species *Peromyscus leucopus* (white-footed mouse) while four other deermouse genomes (*P. maniculatus*, *eremicus*, *californicus*, and *azthecus*) carried the MTD insertion. In the *Arvicolinae* family, all examined species except of *Ondatra zibethicus* (muskrat), carry the same deletion (Fig. 1c and Additional file 1: Fig. S2). Off note is that *Ondatra* is the only species where the conserved AUG codon from the MTD LTR was lost and where we could not identify the syntenic splice acceptor in the exon 2 (Fig. 1b). These three independent losses in *Arvicolinae* and *Neotominae* imply absence of positive selective pressure on maintaining the MTD LTR promoter in *Cricetidae*.

### D6Ertd527e expression in rodent oocytes

**Fig. 2:**
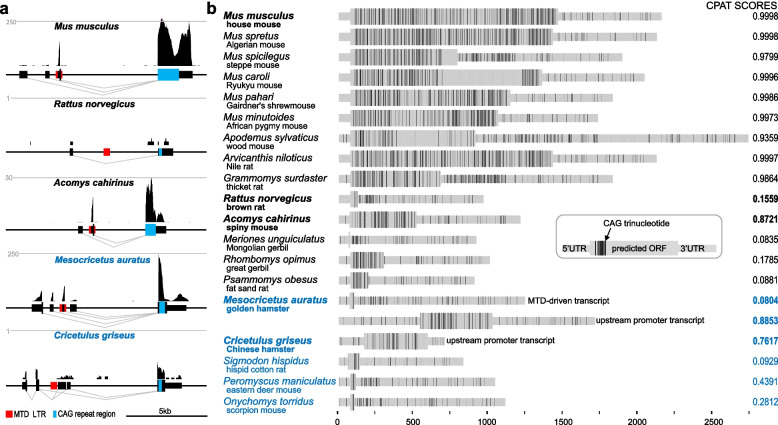
*D6Ertd527e* transcript variability. **a** Variability of exon–intron structure of *D6Ertd527e* transcripts in oocytes of five different rodent species. Shown are modified UCSC genome browser snapshots depicting distribution of RNA-seq reads, level of expression and exon–intron structures inferred from analysis of spliced individual sequence reads. Position of the MTD LTR insert is indicated by red rectangles. Blue rectangles depict regions containing expanded CAG repeats. Full display of repetitive sequences from Repeatmasker is available in Additional file 1: Fig. S2. Dashed lines indicate normalized expression level in CPMs. *Rattus norvegicus* analysis revealed a single spliced read from > 120 million mapped reads from four independent libraries. **b** Distribution of AGC codons in predicted *D6Ertd527e* transcripts in rodent species carrying the MTD LTR insertion. In case of *Cricetulus griseus*, we used the most abundant transcript isoform transcribed from a promoter upstream of the MTD insert. In case of *Rattus norvegicus*, where the locus seems silent, we show a hypothetical transcript spliced between the conserved splice sites (Fig. 1c) to demonstrate that the putative coding sequence starting from the AUG codon in MTD is soon terminated. CPAT score [37] was calculated for predicted coding sequences represented by the thicker part of a transcript scheme. The recommended cut-off for the mouse coding probability for the CPAT release 3.00 was 0.44 [37]

*D6Ertd527e* expression in rodent oocytes could be examined in transcriptomes of five *Muroidea* species. While the maternal transcriptome of *Cricetulus griseus* (Chinese hamster) was newly sequenced, RNA-seq data from *Mus musculus*, *Rattus norvegicus* and *Mesocricetus auratus* (golden hamster) were obtained from the literature [12, 27, 35]. From the *Acomys cahirinus* (spiny mouse) were available only zygotic samples [36], but they allowed for identifying the *D6Ertd527e* transcript. Analysis of RNA-seq data revealed diversity of *D6Ertd527e* expression levels, functional gene elements, and expression (Fig. 2a). The MTD insert has become the main dominant promoter in *Mus musculus*, *Acomys cahirinus*, and *Mesocricetus auratus*. However, in *Mesocricetus auratus*, three additional promoters also yielded considerable amount of *D6Ertd527e* transcripts (Fig. 2a and Additional file 1: Fig. S3).

Although present in the genome, the MTD LTR promoter was essentially inactive in rat and had minimal activity in Chinese hamster oocytes. In rat oocytes, we have found only a single read in the *D6Ertd527e* locus unambiguously coming from a spliced transcript. But it clearly originated from a different promoter than the MTD LTR promoter. In fact, there was no evidence for transcriptional activity of the MTD LTR promoter (Fig. 2a). This is remarkable considering the presence of the MTD promoter and the fact that house mouse and spiny mouse express *D6Ertd527e* well (Fig. 2a)*.*

In Chinese hamster oocytes, *D6Ertd527e* expression was negligible relative to expression in golden hamster oocytes and originated mainly from a promoter upstream of the MTD LTR promoter (Fig. 2a). Taken together, *D6Ertd527e* expression varies greatly in *Muroidea* oocytes and highest observed levels of *D6Ertd527e* expression are supported by the MTD LTR promoter.

### Protein-coding potential of D6Ertd527e

**Fig. 3:**
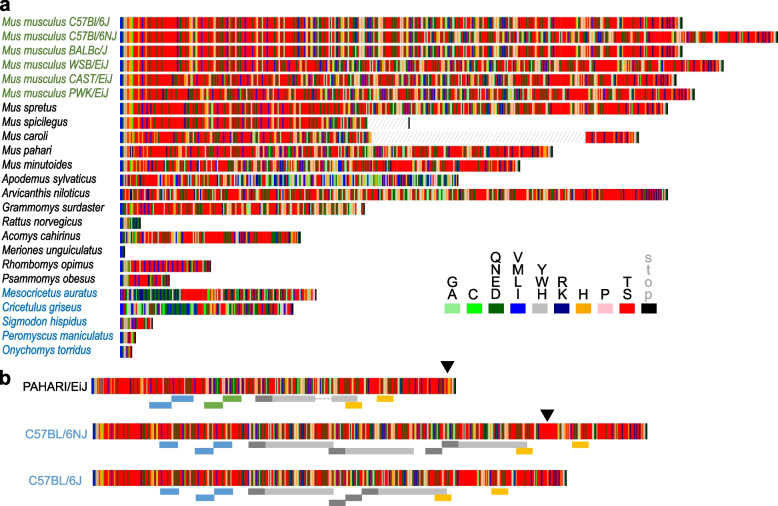
Diversity and composition of predicted *D6Ertd527e* proteins. **a** Aminoacid composition of selected rodents inferred from transcriptome data and predicted MTD-driven *D6Ertd527e* transcripts (Fig. 2b). From the previously analyzed *Mus musculus* inbred strains [12], examples were selected to illustrate variability among the strains. Hatching in *Mus spicilegus* and *Mus caroli* reflects presence of a block of Ns in their genomic DNA. **b** Composition of the coding sequence in the genus Mus shows that expansion of the coding sequence stems partially from CAG repeat expansion (one such a repeat is indicated by black arrowheads) but mostly from sequence duplications of variable lengths (various duplicated segments are depicted by colored rectangles below the protein sequence)

*D6Ertd527e* transcripts originating from the MTD promoter carry predicted coding sequences of variable lengths and amino acid composition (Fig. 3). MTD LTR caries a conserved AUG initiation codon (in fact, it is AUGAUG, Fig. 1a), which likely came with the original insert in the common ancestor of mice and hamsters, but its significance is difficult to interpret. When analyzing nucleotide-exchange rate of MTD LTR nucleotides (Additional file 1: Fig. S4), the first nucleotide of the first codon in AUGAUG sequence appears to have a higher nucleotide exchange rate than other nucleotides. Consequently, 58% of MTD carry at least 1 AUG: ~ 14% of MTD LTRs carry the first AUG, ~ 29% of MTD LTRs have the second AUG present, and 15% of MTD LTRs carry both. Thus, the persistence of AUG in the *D6Ertd527e* MTD LTR might reflect functional significance. However, six examined species lost the entire MTD insert and two have minimal or no expression. Finally, golden hamster produces four *D6Ertd527e* transcripts differing in the 5′ terminal exon and the MTD-driven transcript does have a strong coding potential while one of the other three isoforms has (Fig. 2).

Predicted coding sequences of *D6Ertd527e* transcripts are typically serine-rich as a consequence of (CAG)_n_ repeat expansion in the last exon. The presence of (CAG)_n_ clusters in the *D6Ertd527e* locus in *Cricetidae* species indicates that a short (CAG)_n_ repeat must have been present in the common ancestor of hamsters and mice (Fig. 2). *D6Ertd527e* transcripts have variable (CAG)_n_ repeat distribution but (CAG)_n_ repeats typically locate in the protein coding sequence, especially if the coding sequence is longer. In several rodent genomes, such as in *Mus spicilegus* (steppe mouse), *Apodemus sylvaticus* (wood mouse), and *Grammomys surdaster* (thicket rat), there are large (CAG)_n_ repeat clusters also in predicted 3′ UTRs (Fig. 2B). In the rat genome, the predicted CDS is minimal and most of CAG trinucleotides are downstream of it (Fig. 2b).

The predicted protein-coding sequences of *D6Ertd527e* in extant species reveal dynamic evolution of the protein coding sequence in the *Muroidea* superfamily (Figs. 2b and 3a). There is a highly variable length of the coding sequence even among closely related species (Fig. 2b). Many species (e.g., *Meriones unguiculatus* (Mongolian gerbil), *Rattus norvegicus*, *Onychomus torridus* (scorpion mouse), *Peromyscus maniculaculatus* (eastern deer mouse)) have minimal coding sequences, suggesting that MTD-driven *D6Ertd527e* homologs in these species are not protein coding.

However, in the absence of maternal transcriptome data, it is unclear whether there could be alternative transcripts with longer CDS. This issue is exemplified in Golden hamster where the *D6Ertd527e* locus is well expressed and four different 5′ terminal exons are spliced to the 2^nd^ (last) exon (Fig. 2a). Notably, the MTD-driven transcript coding sequence has a short CDS terminated at the beginning of the 2^nd^ exon and is likely non-coding. Two other transcripts from the *D6Ertd527e* do not carry significant CDS as well but the most upstream promoter drives expression of a putative protein-coding transcript with a reasonably large CDS (Fig. 2).

Reading frames in a (CAG)_n_ repeat encode three possible peptide chains: polyQ, polyS, or polyA. Notably, long *D6Ertd527e* reading frames in *Muridae* species are devoid of frameshifting and generally remain within the polyS frame while their polyQ reading frames are riddled with stop codons. The polyQ reading frame accumulates stop codons naturally because a single point mutation in (CAG)_n_ can form a stop codon only in the polyQ frame (C to T conversion resulting in TAG). In contrast, single point mutations in polyS or polyA frames of (CAG)_n_ are either silent or cause an amino acid change. Consequently, the serine-rich, *D6Ertd527e* CDS among *Muridae* species show high divergence of predicted protein coding sequences (Fig. 3a), which stems from a combination of (CAG)_n_ expansion, duplications/recombination events, and point mutations (Fig. 3b). Variability exists even among laboratory strains, where is particularly remarkable CDS expansion in the C57BL/6NJ strain, which has the longest CDS. C57BL/6NJ is an NIH subline of C57BL/6. It was separated from C57BL/6 J (mouse reference sequence) in 1951 [38] and carries an extra duplicated segment unlike the closely related C57B/6 J or more distant BALB/cJ (Fig. 3a, b).

Deviations from the utilization of the serine reading frame in the predicted protein sequences were found in *Apodemus sylvaticus* and hamsters (Fig. 3a). In *Apodemus*, the polyS frame at the N-terminus is rather short and approximately 2/3 of the protein are not rich in (CAG)_n_ repeats (Fig. 3a). In hamsters, the MTD-driven transcript does not carry a long ORF (golden hamster) or is not expressed at all (Chines hamster). However, both hamsters utilize an upstream promoter driving expression of a transcript with longer CDS. The predicted Chinese hamster *D6Ertd527e* CDS identified in this transcript is relatively long and with a glutamine-rich segment, which would give the predicted encoded protein entirely different properties than exhibit other *D6Ertd527e* homologs in the *Muridae* family (Fig. 3a). However, as mentioned above, expression of *D6Ertd527e* in Chinese hamster oocytes is minimal. This contrasts with golden hamster, where this transcript with protein coding potential is well expressed. The predicted protein carries a glutamine-rich segment at the N-terminus but also a frameshift into the polyS frame in the central part of the predicted protein (Fig. 3a). While significance of this *D6Ertd527e* remains unknown, it provides another example of significant divergence of a potentially encoded protein from the *D6Ertd527e* locus*.*

Taken together, many *Muridae* species evolved relatively long MTD-driven *D6Ertd527e* CDS and their predicted protein sequences considerably diverged from a homopolymeric amino acid sequence that would be encoded by a perfect trinucleotide repeat. Relative absence of frameshifts between polyS, polyQ, and polyA frames of (CAG)_n_ repeats in mice implies that their *D6Ertd527e* homologs might encode serine-rich proteins that are under neutral or positive selection. In contrast, *Cricetidae* did not evolve longer MTD-driven *D6Ertd527e* CDS. Instead, the MTD LTR was repeatedly lost and two *D6Ertd527e* CDS transcripts with predicted longer CDS originate from a different promoter.

### Murine D6Ertd527e protein features

**Fig. 4:**
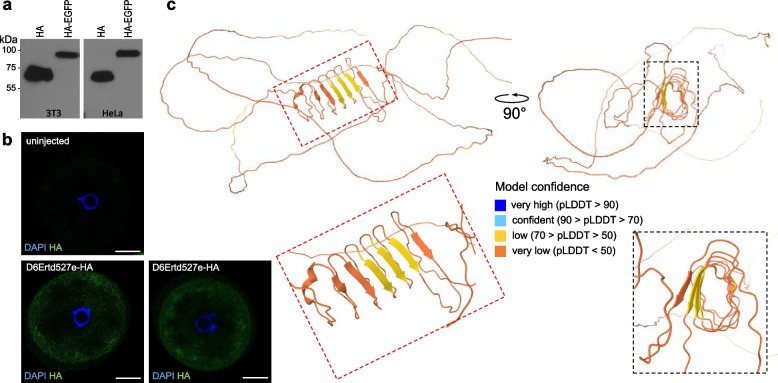
Murine D6Ertd527e protein expression and structure. **a** Ectopically expressed C-terminally tagged D6Ertd527e protein in NIH 3T3 and HeLa cells can be detected by Western blotting. **b** Expression of C-terminally HA-tagged D6Ertd527e in oocytes analyzed by immunofluorescent staining and confocal microscopy. Approximately 100 000 in vitro-transcribed mRNA molecules were microinjected into mouse fully-grown GV oocytes and the protein was visualized by immunofluorescent staining with α–HA antibody (green color). DNA was stained with DAPI (blue color). Size-bar = 20 μm. **c** Hypothetical folding of D6Ertd527e protein predicted by AlphaFold [43]

Murine *D6Ertd527e* is a *bona fide* protein-coding gene, as its protein product was reported in proteomic studies of mouse oocytes [39–42], and six different D6Ertd527e peptides can be identified in released data [41]. Likewise, D6Ertd527e fused to a C-terminal hemagglutinin (HA) tag can be ectopically expressed in cultured mammalian cells and detected as a protein of the expected size by immunoblot analysis (Fig. 4a, Additional file 2). In cultured cells, the *D6Ertd527e* protein diffusely localized to the cytoplasm, with no apparent effect on the expressing cells [12]. Ectopic expression of D6Ertd527e–HA demonstrated that the MTD LTR provides a functional 5′ UTR and translation initiation start and the (CAG)_n_ repeat-derived CDS can be translated into a detectable non-aggregating protein. We also expressed D6Ertd527e-HA protein from mRNA microinjected into the oocyte. We microinjected ~ 100,000 molecules of *D6Ertd527e*-HA mRNA into fully-grown GV oocytes, cultured them for 20 h and stained them with α-HA antibody. Staining appeared stronger in oocyte’s periphery and some denser areas (Fig. 4b). Taken together the CDS gives rise to a stable protein, which does not exhibit strong aggregation propensity.

Recent advances in protein structure predictions enables to build protein models in silico with a good accuracy [43]. Structural prediction of D6Ertd527e revealed a largely unstructured protein with an unusual central beta-sheet barrel (Fig. 4c). However, this central structure was predicted with a low confidence and was not observed in structural predictions of D6Ertd527e homologs in *Mus pahari* (shrew mouse) and *Acomys cahirinus* (spiny mouse, Additional file 1: Fig. S5) suggesting that it is not a conserved functional element of D6Ertd527e. We thus conclude that D6Ertd527e-encoded proteins are typically intrinsically disordered serine rich proteins.

### Functional analysis of D6Ertd527e

**Fig. 5:**
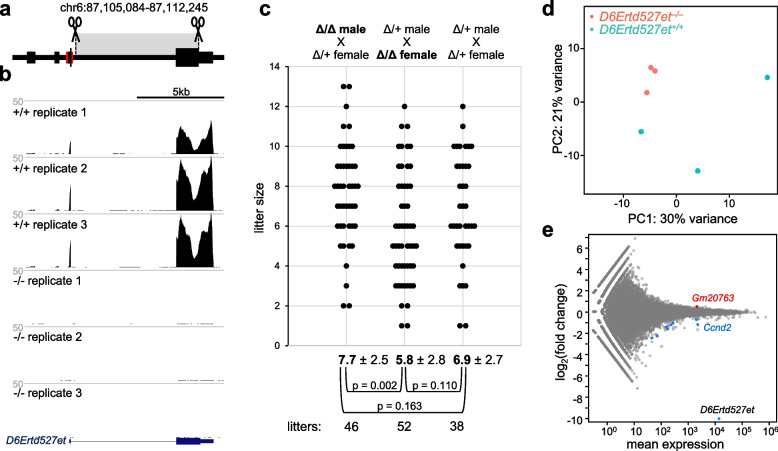
*D6Ertd527e* knock-out analysis. **a** Schematic depiction of positions of designed CRISPR cleavage points. **b** A UCSC browser snapshot of data from RNA-seq libraries from oocytes from three wild-type mouse and three mutants. Low residual signal in the coding sequence in *D6Ertd527e* mutants can be explained by multimapping repetitive reads originating from other loci. **c** Breeding performance of matings with different combinations of genotypes. *p*-values were calculated with two-tailed *t*-tests. **d** PCA analysis of RNA-seq libraries suggests higher variability of wild-type controls and clustering of mutant transcriptomes. **e** MA plot depicting differentially expressed genes in *D6Ertd527e* mutant oocytes. Significantly upregulated and downregulated transcripts are shown in red and blue, respectively. The most abundant significantly changed transcripts were *Gm20763* and *Ccnd2*

To examine biological significance of *D6Ertd527e*, we used CRISPR guided nucleases to generate a mouse deletion model for *D6Ertd527e*. CRISPR-cleavage positions were intended to delete the intron and the coding sequence from the last exon while retaining the MTD LTR and the conserved 3′ UTR sequence (Fig. 5a). We successfully produced mutant mice carrying deletion of the intron and the coding part of the 3′ terminal exon (Additional file 1: Fig. S6), which resulted in the loss of *D6Ertd527e* expression (Fig. 5b). Mutant mice were fertile but had slightly smaller average litter size (Fig. 5c).

The litter size was smaller by approximately one pup but this difference was not significant when we compared all results from mutant and heterozygous females mated with heterozygous males (*p* = 0.110, two-tailed t-test, Fig. 5c). However, inspection of breeding data suggested that litter size has variability affected by lower sizes of the first litters, which is a known phenomenon [44]. When this genotype-independent variability of first litters was removed from the analysis by not including them, the litter size of knock-out and heterozygous females mated to heterozygous males became statistically significant (p = 0.007, two-tailed t-test).

This difference was not accompanied by any remarkable transcriptome change. Transcriptome profiling of mutant oocytes by RNA sequencing showed negligible changes in gene expression (Fig. 5d, e). The most abundant differentially expressed transcripts were *Gm20763* (increased abundance) and *Cyclin D2* (*Ccnd2*, reduced abundance). However, these changes do not seem to be functionally significant. *Gm20763* is an ORR1A2 LTR-driven lncRNA carrying an antisense *Kif1c* pseudogene. This class of maternal lncRNAs would bind *Kif1c* mRNA and trigger endogenous RNAi [45]. However, the *Kif1c* is not among significantly downregulated genes indicating that the observed increase in *Gm20763* level does not affect RNAi-mediated repression of its target. Similarly, significance of reduced mRNA levels of *Ccnd2* in prophase-arrested fully-grown oocytes is questionable as this gene is important for cumulus cells but not the oocytes as shown in the *Ccnd2* knock-out where oocytes lacking CCND2 meiotically mature and develop to the blastocyst stage after fertilization at normal rates [46].

Taken together, absence of *D6Ertd527e* appears to have minor effect on fertility of mutant oocytes while transcriptome changes are minimal and do not provide any explanation for the reduced litter size of *D6Ertd527e*^–*/–*^ females.

## Discussion

**Fig. 6:**
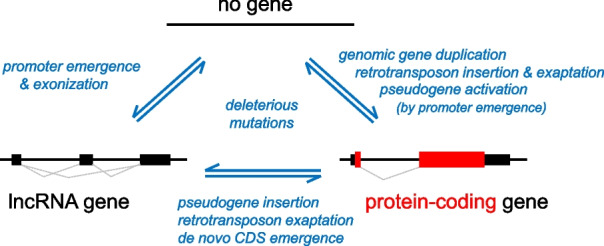
Phases of gene life-cycle during evolution. The upper scheme represents a locus, which does not produce any transcript. Such a locus can give rise to a lncRNA - emergence of a pol II promoter will be sufficient to produce a non-coding transcript. A promoter can emerge from a random sequence or through a solo LTR insertion. The initial transcript at the locus will likely be a lncRNA with variable exon–intron structure as mRNA processing mechanisms will recognize with variable efficiency cryptic splice donors and acceptors as well as poly(A) sites. Such a lncRNA can evolve into a protein-coding gene through recycling a protein-coding sequence from a processed pseudogene inserted into the locus

Here we report evolutionary history and functional analysis of a de novo formed murine gene, which offers an outstanding example of numerous phases of gene emergence, evolution and demise (Fig. 6) on an evolutionary scale of tens of millions of years. The gene *D6Ertd527e* emerged in an intergenic region in the common ancestor of mice and hamsters. The same syntenic locus in porcine and bovine genomes does not produce a known transcript while the human locus carries an MLT1A0 LTR-derived promoter and the first exon of an oocyte-specific unannotated lncRNA that does not share any feature with *D6Ertd527e* and clearly represents an independent evolutionary event [12].

The critical event in the *D6Ertd527e* locus was insertion of an MTD LTR, which provided an oocyte-specific promoter and a full 5′ terminal exon. The locus may have contained or evolved additional promoters, as suggested by alternative upstream transcription start sites in mouse and hamster genomes. However, the initial MTD LTR insertion already contained an AUG codon, which appears as the start codon in most predicted *D6Ertd527e* coding sequences in *Muridae* and its functionality has been validated in the murine *D6Ertd527* (Fig. 4a, b and [12]). The conserved splice donor in the MTD LTR is spliced with a downstream splice acceptor, which presumably evolved from a cryptic splice acceptor. At least there is no evidence outside of murids that this splice acceptor would be a part of another functional transcript.

Evolution of the *D6Ertd527* in mice and hamsters took diverse paths. The MTD LTR driving *D6Ertd527e* expression was lost during *Cricetidae* evolution at least three times due to internal deletions (Additional file 1: Fig. S2). Admittedly, the analysis relied on rodent genome assemblies obtained by different methods and variable quality of assembly. However, existence of the deletion in *Arvicolinae* is supported by presence of the same apparent deletion in four different species. Deletion in a single species of genus *Peromyscus* is supported only indirectly. The *D6Ertd527e* gene region in nine *Peromyscus* species is assembled well. *Peromyscus leucopus*, which lacks the MTD insertion, and its closest sequenced relative *Peromyscus maniculatus* have their genomes (GCA_004664715.2 and GCF_003704035.1) assembled to the chromosomal level, which are higher quality assemblies [47, 48].

The protein-coding capacity of the *D6Ertd527e* transcript expressed from the MTD LTR likely have evolved stochastically. The functional AUG in the original MTD insert would prime evolution of a protein-coding transcript but extreme variability of *D6Ertd527e* coding sequences starting from that MTD AUG suggests that *D6Ertd527e* either initially were lncRNAs or repeatedly evolved into ones. It is important to point out that there is no strict disjunction between pol II-transcribed spliced polyadenylated lncRNAs and protein-coding mRNAs as ribosomes usually scan lncRNAs and might translate their short putative coding sequences [49, 50].

Expansion of simple nucleotide repeats in genomic DNA is a common phenomenon (reviewed in [51]). In a coding sequence, expending nucleotide repeats will give rise to amino acid repeats (homorepeats in case of expanding trinucleotides) in affected proteins. Amino acid homorepeats in proteins are diverse in terms of the amino acid type, length, and biological effect (reviewed in [52, 53]). Particularly (CAG)_n_ trinucleotide repeat expansion translated in the polyQ frame, which would exceed certain thresholds in specific proteins, has been associated with a number of pathologies known as polyglutamine diseases [54]. However, amino acid homorepeats also have physiological roles [53] and trinucleotide repeat expansion offers a mechanism for devolution of homopolymeric intrinsically disordered proteins or their regions. A well-established example is an expansion of a low-complexity alanine-rich sequence during convergent evolution of antifreeze proteins in fish [55, 56]. In any case, combination of trinucleotide repeat expansion combined with larger recombination events and point mutations, which change serine residues (ACG codon) into other aminoacids, offer an interesting model for stochastic evolution of protein coding sequence. A single point mutation in the repeat can convert an ACG codon into a codon encoding one of six other amino acids (Gly, Arg, Cys, Asn, Thr, Ile) but not into a stop codon. Two simultaneous point mutations in a codon further increase potential for amino acid changes while having only 3.7% chance of creating a stop codon and disrupting the evolving CDS. This is consistent with the appearance of murine *D6Ertd527e* CDS where the (CAG)_n_ repeat has been eroded by point mutations and recombinations while pure (CAG)_n_ repeats are restricted to specific regions, which seem to expand independently (Fig. 3a).

At the same time, the polyQ frame appears to be avoided in putative D6Ertd527e proteins translated from the start codon in the MTD LTR-derived exon. The proteins are serine rich, with only one major switch to the alanine-encoding (CAG)_n_ reading frame. As this phenomenon stretches across the entire *Muridae* family, it suggests some positive selection for its maintenance might exist.

As the CDS analysis depends on the quality of genome assembly, it should be pointed out that most of the *D6Ertd527e* locus sequences were assembled without any gaps within the repetitive coding sequence. This is not surprising as the coding (CAG)_n_ repeats are typically eroded by mutations, which facilitates sequence assembly, and perfect (CAG)_n_ repeats in sequences are typically not long enough to interfere with sequencing assembly. The only exceptions are *Mus spicilegus* and *Mus carroli*, which each have a single gap within the assembled *D6Ertd527e* CDS. Furthermore, when transcriptome data were available, their mapping onto the assembled genomes did not reveal any issues with assembled genomic sequences of *D6Ertd527e*.

Did *D6Ertd527e* evolve some function as a protein-coding gene*?* The length and preservation of the CDS translated in the serine frame in the lineage leading to house mouse indicates that *D6Ertd527e* may indeed have some function. This notion is supported by slightly reduced litter size (15–20%) of *D6Ertd527e*^*–/–*^ females. On evolutionary scale, such an effect on reproductive fitness might represent a significant factor. At the same time, we were not able to pinpoint the function of D6Ertd527e protein. Sequence composition and structural analysis suggests that the encoded serine-rich protein is intrinsically disordered. But what biological role could it play?

One interesting example of a low-complexity serine-rich sequence is the Phosvitin (Pv) domain/protein from Vitellogenin, an egg-yolk precursor (reviewed in [57]). One function of Vitellogenin and yolk proteins is antioxidant activity providing protection against oxidative damage [58, 59]. Phosvitin is a serine-rich polypeptide (> 50% serine), which is highly phosphorylated [60], attracts multivalent cations as calcium, magnesium, zinc, and iron [61] and its iron chelation ability was shown to reduce DNA damage [62]. Interestingly, Vitellogenin genes were lost during mammalian evolution during transition from yolk-dependent nourishment toward lactation and placentation [63]. That D6Ertd527e protein could contribute to reduction of oxidative damage and substitute function of Vitellogenin sounds attractive but it is not fully consistent with all data as the level of phosphorylation of *D6Ertd527e* is unclear. Mass-spec analysis supporting murine D6Ertd527e peptides in oocytes detected non-phosphorylated peptides. Likewise, a discrete band of ectopically expressed HA-tagged D6Ertd527e in 3T3 and HeLa cells (Fig. 4a) does not seem to support the notion of a highly phosphorylated D6Ertd527e. In any case, some cytoplasmic function of D6Ertd527e stemming from its biophysical features is a likely one that could purposelessly emerge during evolution of *D6Ertd527e*.

## Conclusions

There is a number of characterized de novo protein-coding genes in vertebrates or elsewhere, which are described in the literature and are of a comparable age or even younger than *D6Ertd527e* (reviewed in [64, 65]). However, uniqueness of *D6Ertd527e* is that its documented evolution makes it an excellent textbook example of stochastic events, which bring into being a transcriptional unit in the genome, which can either evolve into a protein-coding gene, remain, or disappear during evolution.

## Methods

### Animals

Animal experiments were approved by the Institutional Animal Use and Care Committees (Approval no. 58–2015) and were carried out in accordance with the law.

### Oocyte and embryo collection

Fully grown, germinal vesicle (GV)-intact oocytes were obtained from C57Bl/6NCrl mice as described previously [66]. Oocytes were collected and microinjected in M2 medium supplemented with 0.2 mM 3-isobutyl-1-methyl-xanthine (IBMX; Sigma) and cultured in M16 medium (Sigma-Aldrich) supplemented with 0.2 mM 3-isobutyl-1-methyl-xanthine (IBMX; Sigma), at 37 °C in a 5% CO_2_ atmosphere.

### Oocyte microinjection

RNA for injection was diluted in pure water such that 100,000 molecules would be present in 5 picoliters (pl). Microinjections were done using a FemtoJet microinjector (Eppendorf). Femtojet injection pressure was set to maintain injection volume of 5 pl for all microinjections. Reliability of the estimated amount of microinjected molecules was experimentally addressed previously [67]. Injected mouse oocytes were cultured in M16 media (Merck) supplemented with IBMX in 5% CO_2_ at 37 °C for 20 h.

### CRISPR-mediated deletion of D6Ertd527e

The deletion mutant model was produced in the Czech Centre for Phenogenomics at the Institute of Molecular Genetics ASCR using Cas9-mediated deletion of *D6Ertd527e* intron 1 and the protein-coding sequence of exon 2 (Additional file 1: Fig. S6). Sequences of guide RNAs were sgRNA T5 5′-CCTCGAGATGAGCCATCC-3′ and sgRNA E2 5′-CTTAGGAAATCATTCCCA-3′. To produce guide RNAs, synthetic 128 nt guide RNA templates including T7 promoter, 18nt sgRNA, and tracrRNA sequences were amplified using T7 and tracrRNA primers. Guide RNAs were produced in vitro using the Ambion mMESSAGE mMACHINE T7 Transcription Kit and purified using the mirPremier™ microRNA Isolation Kit (Sigma). The Cas9 mRNA was synthesized from pSpCas9-puro plasmid using Ambion mMESSAGE mMACHINE T7 Transcription Kit and purified using the RNeasy Mini kit (Qiagen). A sample for microinjection was prepared by mixing two guide RNAs in water (25 ng/μl for each) together with Cas9 mRNA (100 ng/μl). Five picoliters of the mixture were microinjected into male pronuclei of C57Bl/6 zygotes and transferred into pseudo-pregnant recipient mice. PCR genotyping was performed on tail biopsies from 4-week-old animals. We obtained a positive founder which transmitted the mutant allele to F_1_, and after two generations of breeding with C57Bl/6NCrl animals, the heterozygotes were used for breeding *D6Ertd527e1*^–/–^ animals for phenotype analysis.

For detection of the knock-out allele were used D6Ertd_gen_Fwd5: 5′-CCTGACACTCAAGAGACACGGTCA and D6Ertd_1_Rev: 5′-CACCTTTCTGTGCTTGTGCTGAAC giving a 877 bp (wild-type allele is absent, too long to amplify). For detecting the wild type allele were used D6Ertd_gen_Fwd5 and D6Ertd_MTD_Rev4: 5′-GAACTGCAAGCTGAGGCTCACAAG, yielding a 812 bp product.

### D6Ertd527e expression vector

Full-length mouse *D6Ertd527e* with the C-terminal HA-tag was synthetized by GENEWIZ in pUC57-Kan plasmid. The coding sequence was cleaved out by NheI and EagI and transferred into pSV40 plasmid backbone. EGFP coding sequence was PCR-amplified and inserted into XbaI and EagI restriction sites to produce D6Ertd527e-HA-EGFP fusion. The final constructs were confirmed by sequencing. The plasmids are available from Addgene: pSV40_mD6Ertd527e-HA as #192,222, pSV40_mD6Ertd527e-HA-EGFP as #192,223:

### Cell culture and transfection

Mouse NIH 3T3 cells were maintained in DMEM (Sigma-Aldrich) supplemented with 10% fetal calf serum (Sigma-Aldrich), penicillin (100 U/ml; Invitrogen), and streptomycin (100 μg/ml; Invitrogen) at 37 °C and 5% CO_2_ atmosphere.

For transfection, the cells were plated on a 24-well plate, grown to 80% density, and transfected with 1 μg plasmid DNA using the Lipofectamine 3000 (ThermoFisher Scientific) according to the manufacturer’s protocol. The cells were collected for analysis 48 h post-transfection.

### Western blotting

Transfected NIH 3T3 cells were washed with PBS and lysed in RIPA buffer (50 mM Tris, pH 7.5, 150 mM NaCl, 1 mM EDTA, 1 mM EGTA, 1% NP-40 (Igepal CA-630), 0.5% Na-deoxycholate, 0.1% SDS) supplemented with 1 × protease inhibitor cocktail set (Millipore). Proteins were separated on 10% polyacrylamide gel and transferred onto a PVFD membrane (Millipore). Anti-HA primary antibody (High affinity, #11867423001, Roche, dilution 1:1000) and HRP-conjugated goat anti-Rat secondary antibody (#31470, ThermoFisher Scientific, dilution 1:50,000) were used for signal detection with SuperSignal West Femto Chemiluminescent Substrate (Pierce).

### RNA sequencing

Total RNA was extracted from 25 wild-type or *D6Ertd527e1*^–/–^ fully-grown oocytes from 8–10-week-old animals using PicoPure RNA Isolation Kit with on-column genomic DNA digestion according to the manufacturer’s instruction (Qiagen). RNA-Seq libraries were constructed using the Ovation RNA-Seq system V2 (NuGEN) followed by Ovation Ultralow Library system (DR Multiplex System, NuGEN). RNA-Seq libraries were pooled and sequenced using 65-nt single-end-sequencing using Illumina HiSeq. *D6Ertd527e1*^−/−^ oocyte sequencing data were deposited in GEO (https://www.ncbi.nlm.nih.gov/geo/) as GSE213820. *Cricetulus griseus* oocyte sequencing data were also deposited under GSE213820. Remaining RNA-seq data were published previously and were obtained from GEO database: *Mus musculus* data accession: GSE116771 [27], *Mesocricetus auratus* data accession: GSE116771 [12] and GSE169528 [33], *Rattus norvegicus* data accession: GSE137562 [35], and *Acomys cahirinus* data accession: PRJNA436818 [36].

### RNA-seq mapping and expression analysis

All RNA-seq data were mapped onto indexed *Mus musculus* (mm10, GCA_000001635.2), *Rattus norvegicus* (rn7, GCA_015227675.2), *Acomys cahirinus* (AcoCah_v1_BIUU, GCA_004027535.1), *Mesocricetus auratus* (MesAur1.0, GCA_000349665.1), and *Cricetulus griseus* (criGriChoV2, GCA_900186095.1) genomes susing STAR 2.5.3a [68] as previously described [27]. Read mapping coverage was visualized in the UCSC Genome Browser by constructing bigWig tracks using the UCSC tools [69]. Differential expression analysis was done in R software environment [70] using DESeq2 package [71] as previously described [27].

## Supplementary Information

**Additional file 1: Figs. S1-S5. Fig. S1.** Additional data on D6Ertd527e gene and its expression. **Fig. S2.** Analysis of deletions in genomic sequences of D6Ertd527e in Cricetidae. **Fig. S3.** Expression of D6Ertd527e in different rodents. **Fig. S4.** Nucleotide exchange rates along the MTD LTR. **Fig. S5.** D6Ertd527e appears to encode an intrinsically disordered protein. **Fig. S6.** Production of D6Ertd527e mutant allele.

**Additional file 2.** Uncropped western blot image used for Fig. 4a.

## Data Availability

Expression data were deposited in the GEO database as GSE213820 [72], and plasmids are available through Addgene (pSV40_mD6Ertd527e-HA as #192,222, and pSV40_mD6Ertd527e-HA-EGFP as #192,223); the *D6Ertd527e* deletion mutant mouse line has been archived and is available upon request as a frozen sperm sample.

## Abbreviations

3T3: 3-Day transfer, inoculum 3 × 10^5^ cells
CDS: Coding sequence
CPAT: Coding-Potential Assessment Tool
CRISPR: Clustered regularly interspaced short palindromic repeats
DMEM: Dulbecco’s Modified Eagle Medium
DNA: Deoxyribonucleic acid
EDTA: Ethylene diamine tetraacetic acid
EGTA: Ethylene glycol tetraacetic acid
GV: Germinal vesicle
HA: Hemagglutinin
IBMX: 3-Isobutyl-1-methyl-xanthine
LINE: Long interspersed element
LTR: Long terminal repeat
lncRNA: Long non-coding RNA
MaLR: Mammalian apparent LTR retrotransposon
MLT: Mammalian LTR retrotransposon (retrotransposon family)
MT: Mouse transcript (retrotransposon family)
MTC: Mouse transcript C subfamily
MTD: Mouse transcript D subfamily
MYA: Million years ago
NIH: National Institutes of Health
ORR: Origin-region repeat (retrotransposon family)
PBS: Phosphate-buffered saline
PCA: Primary component analysis
RIPA: Radioimmunoprecipitation assay buffer
RNA: Ribonucleic acid
sgRNA: Single guide RNA
SINE: Short interspersed element
TE: Transposable element
UCSC: University of California Santa Cruz
UTR: Untranslated region
