# Supplementary material for: De novo emergence, existence, and demise of a protein-coding gene in murids
Source: BMC Biol. 2022 Dec 8;20:272. doi: 10.1186/s12915-022-01470-5 (PMC9733328; doi:10.1186/s12915-022-01470-5)
Supplement: Supplementary file 1 — Additional file 1: Figs. S1-S5. Fig. S1. Additional data on D6Ertd527e gene and its expression. Fig. S2. Analysis of deletions in genomic sequences of D6Ertd527e in Cricetidae. Fig. S3. Expression of D6Ertd527e in different rodents. Fig. S4. Nucleotide exchange rates along the MTD LTR. Fig. S5. D6Ertd527e appears to encode an intrinsically disordered protein. Fig. S6. Production of D6Ertd527e mutant allele. [file 12915_2022_1470_MOESM1_ESM.pdf]

Additional File S1 – Supplementary figures

***De novo* emergence, existence, and demise of a protein-coding gene in murids**

Jan Petrzilek, Josef Pasulka, Radek Malik, Filip Horvat, Shubhangini Kataruka, Helena Fulka, and Petr Svoboda

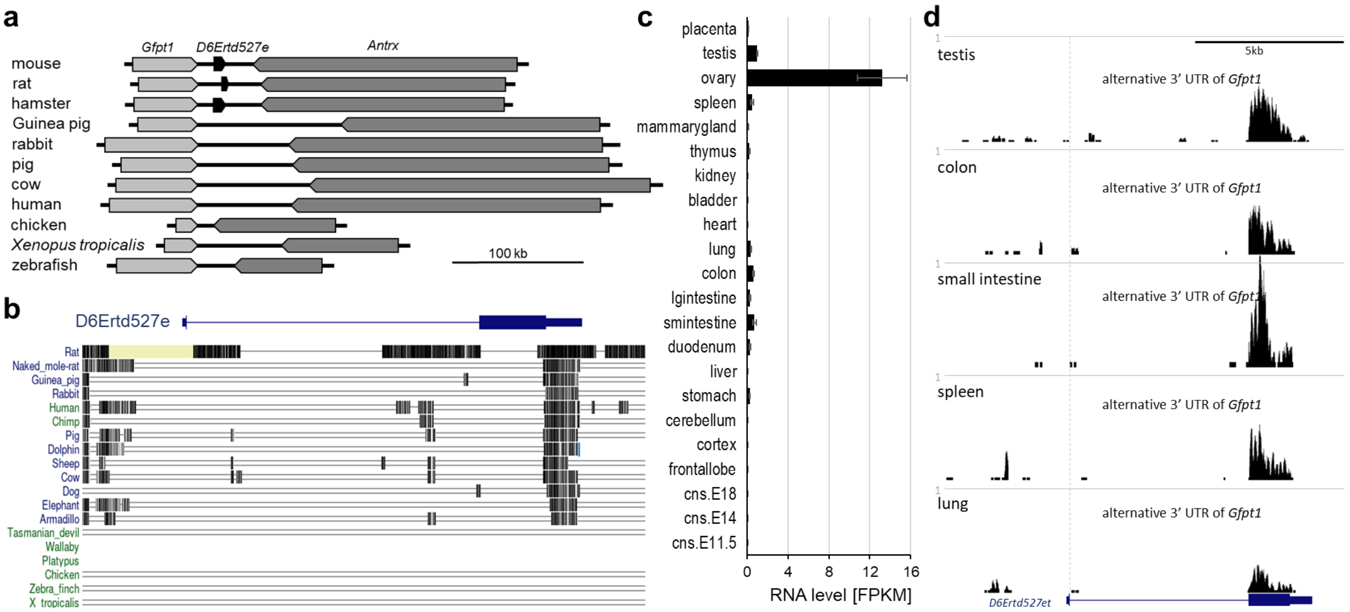

Figure S1. Additional data on *D6Ertd527e* gene and its expression. (**a**) Schematic depiction of synteny of the *Gfpt1*-*Antrx* locus. Transcribed (predicted transcribed) regions of *Gfpt1*and *Antrx* are depicted as grey rectangle arrows whose orientation indicates transcriptional direction. Similarly, *D6Ertd527e* is depicted as a black rectangle arrow. The schemes were generated according to annotated genome sequences of the selected species displayed in the UCSC Genome Browser. (**b**) UCSC Browser snapshot showing the conservation track for a selected list of species. (**c**) *D6Ertd527e* expression in mouse tissues. Expression was estimated as FPKM from the ENCODE polyA RNA NGS mouse tissue raw data (GSE49417 (27)). (**d**) UCSC Browser snapshots of expression profiles of *D6Ertd527e* in selected tissues from the panel c.

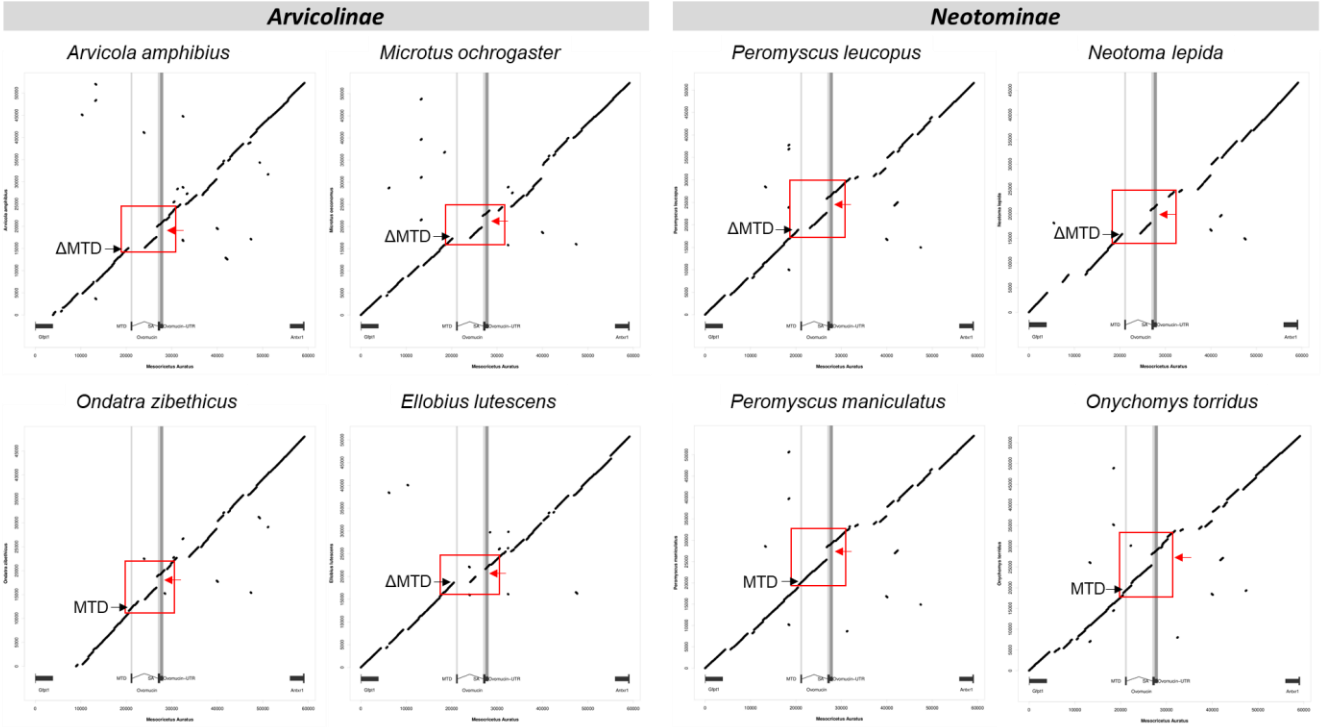

Figure S2. Analysis of deletions in genomic sequences of *D6Ertd527e* in *Cricetidae*. Intergenic sequences between *Gfpt1* and *Antrx1,* including their last exons, were extracted from available genomic sequences of rodent species. Then sequences of *Mesocricetus auratus* and *Mus musculus* were compared pairwise with all other genomic sequences. Shown above are comparisons of *Mesocricetus auratus* genomic sequences with *Arvicolinae* and *Neotominae* species, some of which carry MTD LTR deletions (labeled ΔMTD) and some have the MTD LTR insertion present. Red arrows point to a sequence insertion just upstream of the last *D6Ertd527e* exon. This insertion appears to originate from the common ancestor of *Arvicolinae* and *Neotominae* after the split from *Cricetinae*. More detailed sequence analysis suggests that *Arvicolinae* carry a common deletion while MTD LTR deletions in *Peromyscus* and *Neotoma* represent two additional independent events.

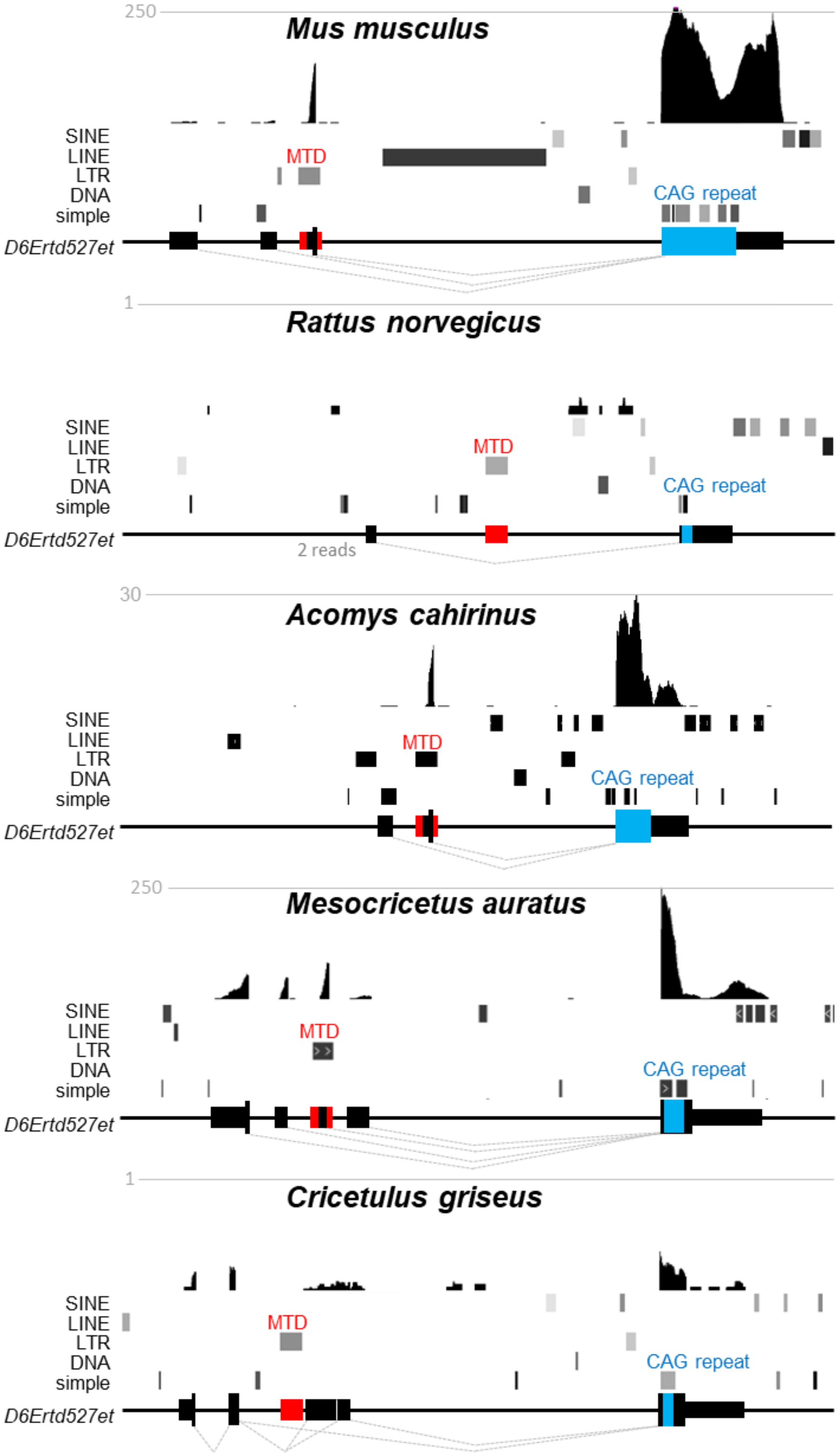

Figure S3. Expression of *D6Ertd527e* in different rodents. This is expanded Fig. 2A with Repeatmasker annotation of repetitive elements (SINE., LINE, LTR, DNA and simple repeats). Positions of the *D6Ertd527e* MTD LTR insert and the $(CAG)_n$ simple repeat are indicated.

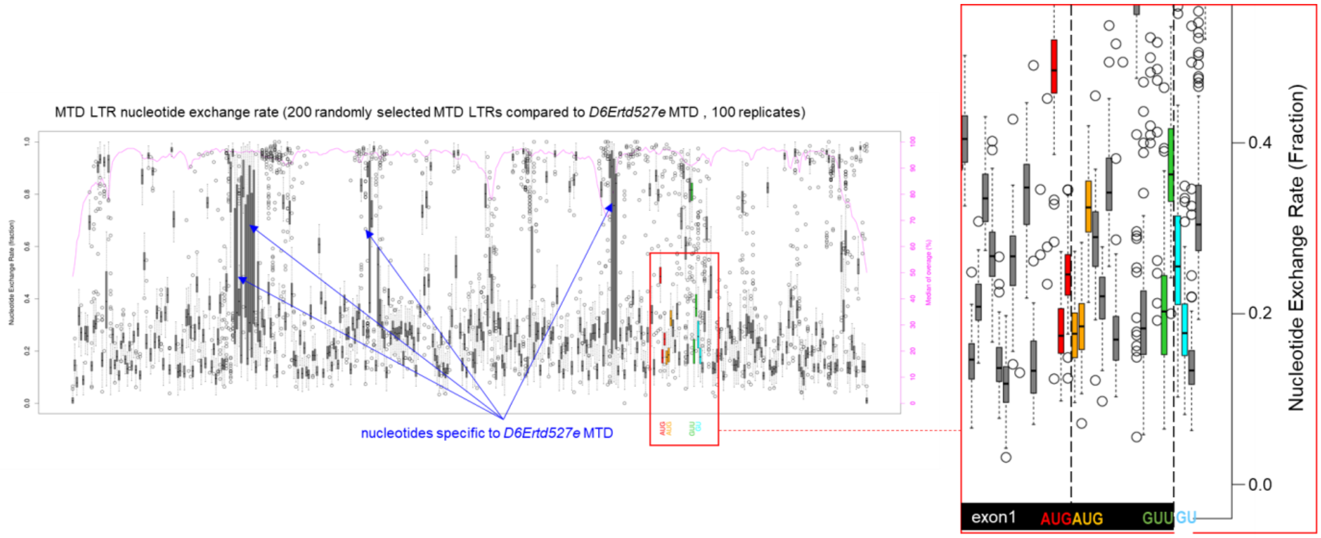

Figure S4. Nucleotide exchange rates along the MTD LTR. *D6Ertd527e* LTR was 10x aligned with 200 randomly selected MTD LTRs from a list of ~13000 MTD LTRs recovered from the mouse genome and nucleotide exchange rate for each nucleotide was calculated for aligned nucleotides. Values for individual nucleotides are depicted as box plots. Red and orange boxplots depict AUGAUG sequence, GU nucleotides of the splice donor are shown in turquoise color**.**

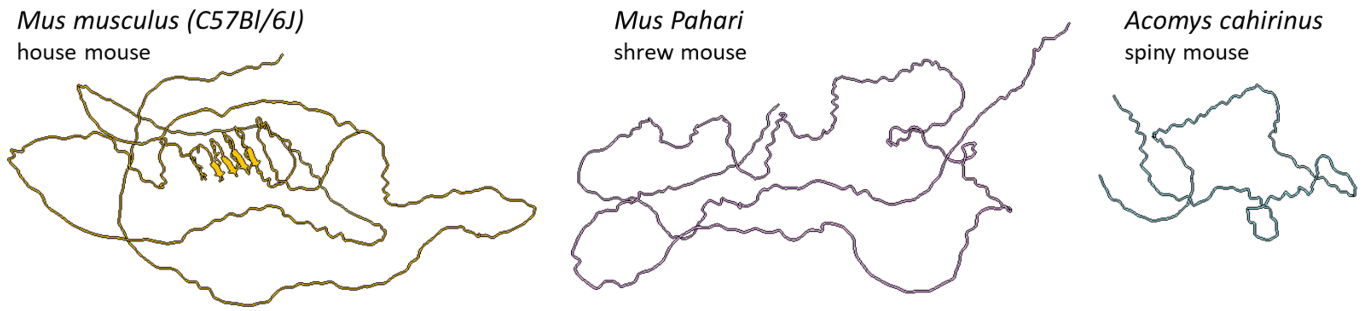

Figure S5. *D6Ertd527e* appears to encode an intrinsically disordered protein. Shown are structural prediction by AlphaFold (36) for predicted coding sequences from the three species.

a

b

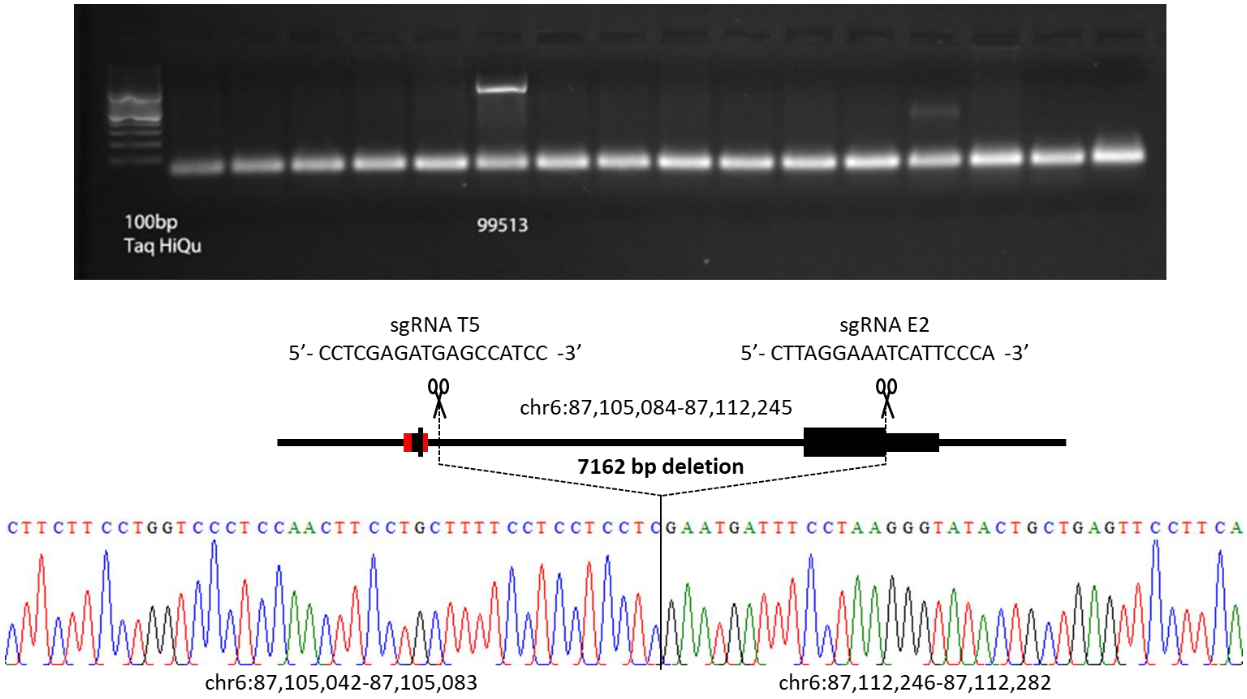

Figure S6. Production of *D6Ertd527e* mutant allele. (**a**) genotyping of F0 animals by PCR. The 6th sample lane (internally labeled 99513) shows *D6Ertd527e* deletion, which was confirmed by Sanger sequencing of the cloned PCR product (the chromatogram depicted in (**b**)).
