# Supplementary material for: De novo emergence, existence, and demise of a protein-coding gene in murids
Source: BMC Biol. 2022 Dec 8;20:272. doi: 10.1186/s12915-022-01470-5 (PMC9733328; doi:10.1186/s12915-022-01470-5)
Supplement: Supplementary file 2 — Additional file 2. Uncropped western blot image used for Fig. 4a. [file 12915_2022_1470_MOESM2_ESM.pdf]

Additional File S2 – Uncropped blot image

De novo emergence, existence, and demise of a protein-coding gene in murids

Jan Petrzilek, Josef Pasulka, Radek Malik, Filip Horvat, Shubhangini Kataruka, Helena Fulka, and Petr Svoboda

- western blot used for Fig. 4A panels
- original scan

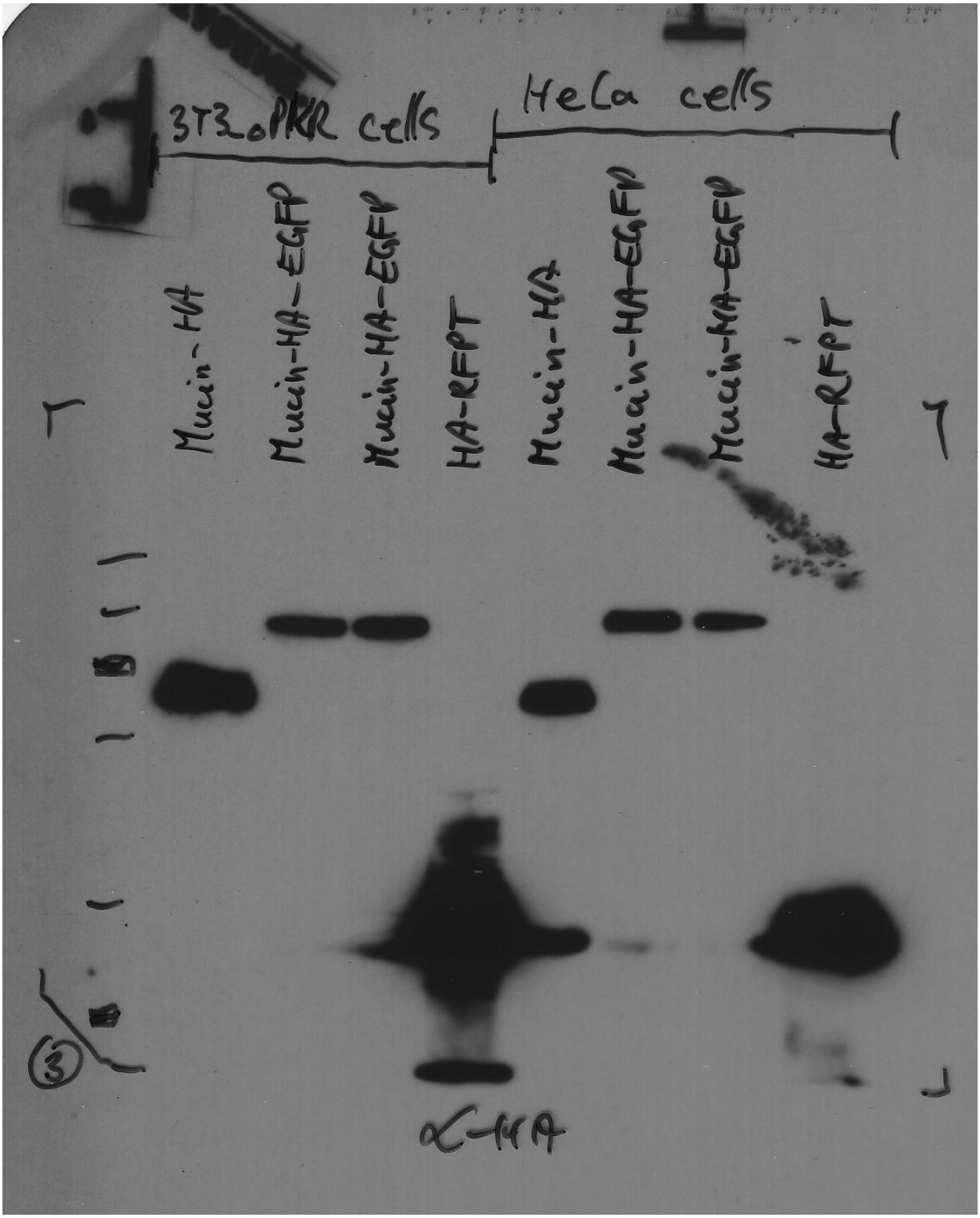

- western blot used for Fig. 4A panels
- annotated scan

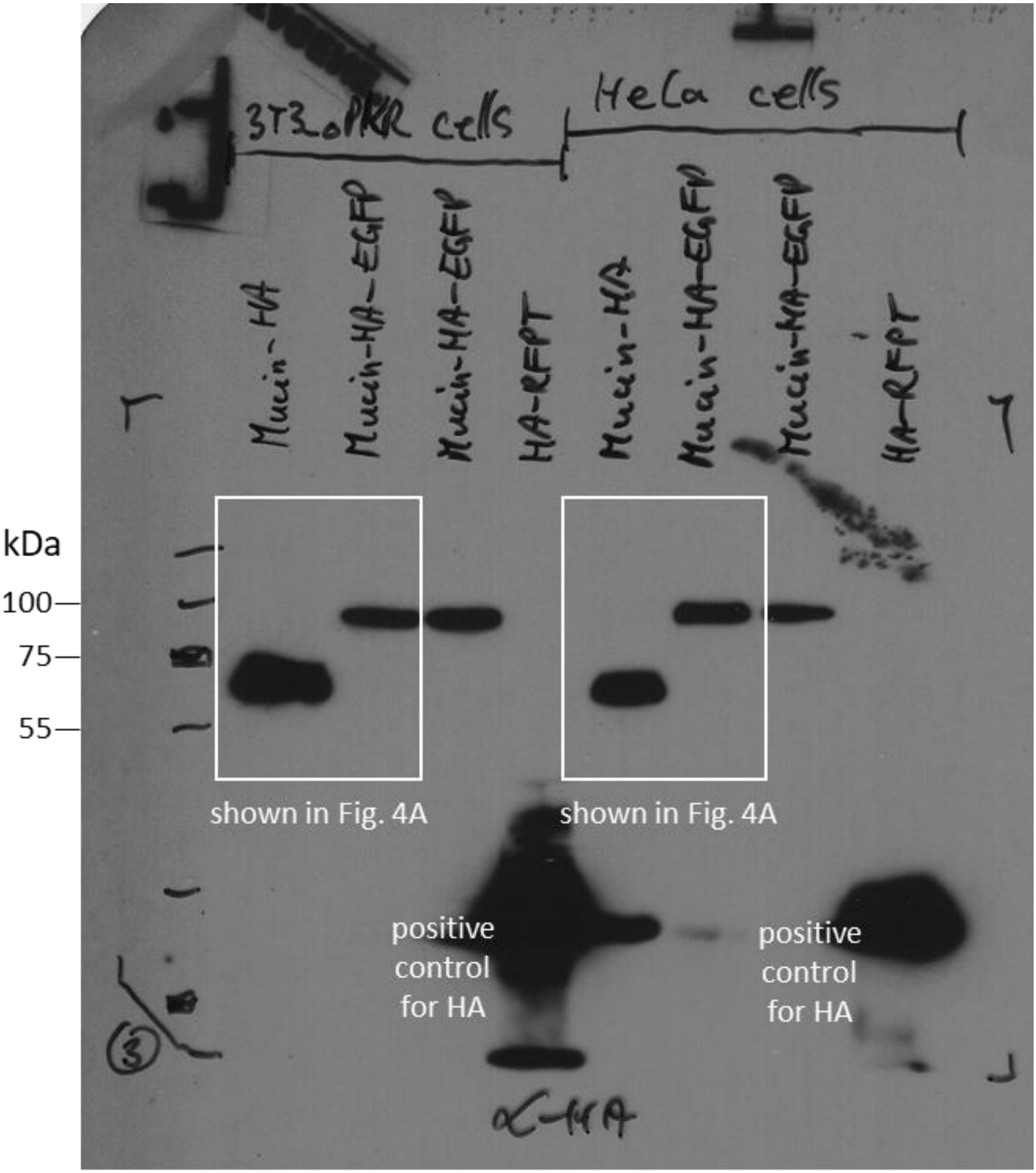

D6Ertd527e is labeled Mucin for historical & practical reasons (it was originally annotated as Mucin-21 and it is easy to remember)
